# Lower Energy-Adjusted Nutrient Intakes Occur Among Food Energy Under-Reporters With Poor Mental Health

**DOI:** 10.3389/fnut.2022.833354

**Published:** 2022-08-08

**Authors:** Karen M. Davison, Vanessa Araujo Almeida, Lovedeep Gondara

**Affiliations:** ^1^Health Science, Kwantlen Polytechnic University, Richmond, BC, Canada; ^2^College of Tropical Agriculture & Human Resources, University of Hawai‘i at Mānoa, Honolulu, HI, United States; ^3^Computing Science, Simon Fraser University, Burnaby, BC, Canada

**Keywords:** mental health, under-reporting, nutrition, measurement error, dietary intakes

## Abstract

**Background:**

Food energy under-reporting is differentially distributed among populations. Currently, little is known about how mental health state may affect energy-adjusted nutrient intakes among food energy under-reporters.

**Methods:**

Stratified analysis of energy-adjusted nutrient intake by mental health (poor vs. good) and age/sex was conducted using data from Canadian Community Health Survey (CCHS) respondents (14–70 years; *n* = 8,233) who were deemed as under-reporters based on Goldberg's cutoffs.

**Results:**

Most were experiencing good mental health (95.2%). Among those reporting poor mental health, significantly lower energy-adjusted nutrient intakes tended to be found for fiber, protein, vitamins A, B_2_, B_3_, B_6_, B_9_, B_12_, C, and D, and calcium, potassium, and zinc (probability measures (*p*) < 0.05). For women (51–70 years), all micronutrient intakes, except iron, were significantly lower among those reporting poor mental health (*p* < 0.05). For men (31–50 years), B vitamin and most mineral intakes, except sodium, were significantly lower among those reporting poor mental health (*p* < 0.05). Among women (31–50 years) who reported poor mental health, higher energy-adjusted intakes were reported for vitamin B_9_ and phosphorus (*p* < 0.05).

**Conclusions:**

Among food energy under-reporters, poor mental health tends to lower the report of specific energy-adjusted nutrient intakes that include ones critical for mental health. Future research is needed to discern if these differences may be attributed to deviations in the accurate reports of food intakes, measurement errors, or mental health states.

## Introduction

A longstanding criticism of self-reported dietary intake data is the underestimation of dietary energy intake (EI) in relation to requirements, commonly referred to as food energy under-reporting (1, 2). This measurement issue that appears to occur non-randomly (1–3) can lead to an inaccurate assessment of the relationships between diet and health (4, 5). Adjustments for EI in the evaluation of nutrient intakes may produce more valid findings as it controls for confounding and removes extraneous variation resulting from factors such as metabolic efficiency (6). A recent study reported that estimates of EI in the 2015 Canadian Community Health Survey (CCHS) were lower than those reported in 2004. The authors suggested that increased misreporting of dietary intake may explain part of this difference (7).

Food energy under-reporting is differentially distributed among populations. Factors associated with food energy under-reporting include female sex, older age, income, body weight status and history, diet composition (e.g., macronutrients), eating behaviors, social desirability, body image, and physical activity (8–10). These characteristics, which contribute to differences in nutrient analysis results, have not been reported in those identified as food energy under-reporters and plausible reporters. Furthermore, although behavior-related issues are relevant, studies of mental health state at the time of dietary intake data collection and its potential effects on nutrient analysis results, particularly among food energy under-reporters, have not been investigated.

The limited research related to mental health state and food energy under-reporting has mainly focused on individuals with a diagnosed condition. A small study, which compared food energy under-reporting in women with schizophrenia and controls, found that food energy under-reporting was more prevalent among those with the mental health condition (77%) vs. those without (50%) (11). In another study that examined individuals with mood disorders, it was found that food energy under-reporting was associated with diet quality, a history of weight change after taking psychiatric medication, and female sex (12). Depending on the type of regression models analyzed, women with probable major depressive episodes (13) or individuals with prior depression diagnosis (14) may have increased odds of food energy under-reporting. Further research is needed about those who report poor mental health, not necessarily those with a diagnosed condition, as this state of mind, which can impact overall functioning, is more common among different populations (15).

To help address gaps in knowledge about the effects of mental health state on nutrient intake analysis results among food energy under-reporters, data from a large, national sample from the CCHS were analyzed. The objective of the analysis was to examine if there are differences in energy-adjusted nutrient intakes among food energy under-reporters experiencing good and poor mental health by age and sex categories. It is hypothesized that the energy-adjusted nutrient intakes among food energy under-reporters will be significantly lower among those experiencing poor mental health when compared to those who report good mental health. The results from the analysis of this national survey may help to determine if mental health state is a factor to account for in studies that include dietary intakes.

## Methods

### Sample of Food Energy Under-Reporters

The sample was derived from Statistics Canada's CCHS – Cycle 2.2 (2004), which provides the only Canadian national data to date that includes both detailed nutrient intake data and a measure of mental health (16). This survey included 35,107 respondents who were living in private residences in all of Canada's 10 provinces. It excluded full-time members of the Canadian Forces and individuals who lived on First Nation Reserves or Crown Lands, in prisons or care facilities, or in some remote areas due to resource limitations or that the health services delivered differ from the general population. Approval for the use of the de-identified dataset was granted by Statistics Canada. All data were vetted by a Statistics Canada analyst prior to release to ensure that respondent privacy was maintained. Institutional Review Board ethics approval was not required.

The sample included CCHS respondents between the age of 14 and 70 years (22,709) who were considered food energy under-reporters as defined by Goldberg's cutoffs for EI-to-basal metabolic rate (BMR) (5). EI plausibility was based on the ratio of self-reported EI from 24-h dietary intake recalls (EIrep) to BMR. Subjects with an EIrep:BMR ratio less than 1.36 were categorized as under-reporters (2). Estimated energy requirements (EERs) (17) were based on respondents' sex, age, self-reported physical activity level, and the self-reported or measured height and weight. The physical activity coefficients used in the EER equation were based on three levels: active, moderately active, or inactive (16).

Energy under-reporting is an important challenge in nutrition epidemiology as it affects the estimation of EI and consequently of other nutrients, which then may lead to a mis-estimation of nutrient inadequacy and bias in the associations between diet and diseases. Given that key characteristics of under-reporters are being women, younger age, and having non-favorable self-reported health perception status (14), the focus of this study was on characterizing energy-adjusted nutrient intakes in energy under-reporters by sex, age, and mental health state. This would enable quantification of the problem, identification of key nutrient intakes that are impacted, and help to identify strategies of how energy under-reporting may be mitigated in future studies.

### Dietary Intake

Dietary intake data were based on 24-h dietary intake recalls that were conducted in-person and included the use of the multi-pass method. For a subset of CCHS respondents, a follow-up 24-h recall was done by telephone between 3 and 10 days after the first interview and this data helped to adjust for day-to-day variability. Energy-adjusted nutrient intakes were derived using the density method where values are reported per 1,000 kcal (18). The Canadian Nutrient File (CNF) was used as the nutrient analysis database. The CNF only had complete values of vitamin E (alpha-tocopherol) for 46% of the foods; therefore, vitamin E intakes were not reported.

### Perceived Mental Health

Perceived mental health, a variable that captures the various dimensions of mental health experiences, was used to stratify the sample by mental health status. The variable is based on responses to the question “How would you say your mental health is: excellent? very good? good? fair? poor?”. The variable was dichotomized as poor mental health (poor/fair responses) and good mental health (good/very good/excellent responses) as has been commonly done in various studies (19–21). Perceived mental health is an indicator for some forms of mental disorder, mental or emotional problems, or distress (22, 23). It has been associated with mental morbidity measures, such as non-specific psychological distress, depressive symptoms, activity limitations, and physical and emotional role functioning (24–27). A recent epigenome-wide association study (EWAS) assessed the predictive value of methylation beta values of EWAS that identified CpGs (5'-C-phosphate-G-3') for incidence of depressive symptoms in later life and found that subjective mental health and hypomethylation at cg27115863 are predictive of depressive symptoms, which are thought to be due to activation of the inflammatory signaling pathway (28).

### Stratified Analysis

For those who were food energy under-reporters, stratified analysis was conducted according to perceived mental health and sex/age categories (14–19, 20–30, 31–50, and 51–70 years). The secured data were analyzed in the Statistics Canada Research Data Center at the University of British Columbia using SAS (version 9.1, 2003, SAS Institute) and Software for Intake Distribution Estimation in IML language (SIDE-IML, version 1.11, 2001, Iowa State University). Survey weights provided by Statistics Canada were incorporated into the calculations to provide national representation, and the bootstrap re-sampling technique was used (16). Nutrient intake values were stratified by age/sex categories and reported using the median and inter-quartile range. Given that the normality assumption is untenable for most nutrient intake distributions (29), statistical comparisons by mental health status within age/sex categories were done using Mann-Whitney *U* tests.

## Results

Of those who participated in the CCHS, between 14 and 70 years (8,233/22,709), 36.3% were considered as food energy under-reporters and formed the basis of the sample used in this investigation. Based on weighted frequencies, 8.9% were between 14 and 19 years, 21.2% were between 20 and 30 years, 41.8% were between 31 and 50 years, and 28.0% were between 51 and 70 years. Within this sample (*n* = 8,233), 95.2% reported good mental health and 51.3% were women.

### Energy, Fiber, and Macronutrients

Among men between 31 and 50 years, energy-adjusted fiber and protein intakes were significantly lower in those reporting poor mental health state (probability measures (*p*) < 0.05; Supplementary Figure S1); conversely, carbohydrate intakes were significantly higher among those reporting poor mental health (Supplementary Figure S1a). For women, significantly lower intakes for protein (31–50 years) and fiber (31–70 years) were reported among those experiencing poor mental health (*p* < 0.05; Supplementary Figure S1b).

### Micronutrients

Among men 20–30 years who reported poor mental health, significantly lower energy-adjusted intakes for vitamins B_2_ and C were found (*p* < 0.05; Supplementary Figure S2a). Similar results were found for intakes of all B vitamins (*p* < 0.05) for men between 31 and 50 years (Supplementary Figure S2b) and vitamins A and D (*p* < 0.05) for men between 51 and 70 years (Supplementary Figure S2c). Among women between 14 and 19 years, energy-adjusted vitamin A intakes were lower among those with poor mental health (Supplementary Figure S2d). Across other age groups for women, energy-adjusted vitamin B_6_ and C intakes (20–30 years; *p* < 0.05; Supplementary Figure S2d) and intakes of vitamins A and B_3_ (31–50 years; *p* < 0.05) were significantly lower among those reporting poor mental health (Supplementary Figure S2e). For women between 51 and 70 years and reporting poor mental health, all vitamin intakes (*p* < 0.05) were significantly lower as compared to those reporting good mental health (Supplementary Figure S2f). Interestingly, among women between 31 and 50 years, vitamin B_9_ intakes were significantly higher among the group with poor mental health (*p* < 0.05; Supplementary Figure S2e).

For mineral intakes, several significant differences by mental health state were also found. Among men 20–30 years, significantly lower energy-adjusted intakes of calcium and zinc were found for those reporting poor mental health (Supplementary Figure S3a). Among men 31–50 years, similar results were indicated for all minerals except sodium (Supplementary Figures S3b,c). For men between 51 and 70 years, calcium intakes were significantly lower among those reporting poor mental health (*p* < 0.05; Supplementary Figure S3a). Among women, significantly lower intakes of energy-adjusted calcium were found for those between 20 and 30 years (Supplementary Figure S3d), and lower calcium, phosphorus, potassium, and sodium intakes were found for those between 51 and 70 years (Supplementary Figure S3e). Among women of 51–70 years, magnesium and zinc intakes were also significantly lower among those reporting poor mental health (*p* < 0.05; Supplementary Figure S3f).

Overall, reported energy-adjusted nutrient intake differences tended to be significantly lower in those reporting poor mental health. Exceptions to this included reported carbohydrate intakes in men 31–50 years as well as vitamin B_9_ and phosphorus in women 31–50 years, where energy-adjusted nutrient intakes were significantly higher among those reporting poor mental health.

## Discussion

Given that most energy-adjusted nutrient intakes were significantly lower among most groups reporting poor mental health, our hypothesis that significantly lower energy-adjusted nutrient intakes would be observed among those with poor mental health was supported. This, however, was not the case for carbohydrate intakes among men 31–50 years, as well as vitamin B_9_ and phosphorus intakes among women 31–50 years, where significantly higher intakes were reported among those reporting poor mental health. Poor mental health state appeared to lower reported energy-adjusted nutrient intakes for protein, fiber, most of the B vitamins, and the majority of minerals, particularly among women and those between 31 and 70 years.

Although it appears that mental health state significantly impacts the report of energy-adjusted nutrient intakes, it is unclear whether those reporting poor mental health state are more prone to under-report food intakes due to reasons such as impairments in recall of food intake (30) or that they are simply consuming less food. In a study, which explored perceived mental health and dietary intakes in the same dataset analyzed for this study, it was reported that those reporting poorer mental health consumed diets of lower quality based on the Canadian Healthy Eating Index (20). In another study, it was indicated that intakes of vitamins B_1_, B_2_, B_6_, B_9_, B_12_, phosphorus, and zinc were significantly lower among individuals with verified mood disorders when compared to a healthy population sample (31). Individuals with poor mental health status who are taking psychiatric medications may experience alterations in usual dietary intakes (32), which could contribute to differences in nutrient intake by mental health state. This would suggest that during data collection, mental health state and medication use should be accounted for and validation approaches, such as the multi-pass method, should be used to help to ensure the reliability of the recorded information. Given the potential impact that mental health state has on reporting of energy-adjusted nutrient intakes, it is questioned whether the results of studies that indicate differences in the reporting of dietary patterns and their associations with mental health outcomes are accurate (33). Our results have highlighted issues related to processes that may cause people to under-report their food intakes. Thus, multidisciplinary approaches, that could include psychology and pathophysiology, are needed to advance the understanding of mental health state and the under-reporting of dietary intake (34).

The findings of significantly higher intakes of carbohydrates among men 31–50 years, as well as vitamin B_9_ and phosphorus among women 31–50 years who reported poor mental health, were surprising. Results of observational studies indicate that recurrent hypoglycemia is associated with poor mental health (35) and this may contribute to increased cravings for carbohydrates and intakes of the macronutrient. Previous studies have shown a positive association between the consumption of soft drinks, which contain high levels of phosphate additives and mental health concerns (36). Intakes of foods with high amounts of folate, have been reported to improve mental health and mood (37). Individuals who are experiencing poor mental health and trying to improve their symptoms may increase intakes of foods which are rich sources of folate.

### Implications

The findings of this study are consistent with others that suggest that energy under-reporting is an issue in research that examines trends in food intakes (38). In particular, our results suggest that dietary intake assessments should utilize the most accurate methods to assess dietary exposures and account for mental health state that is measured by valid tools. If mental health improvements are part of a dietary intervention's goals, particular attention should be made to ensure foods, which are sources of nutrients critical to mental health, such as the omega 3 fatty acids, folate, and iron (39), are accurately recorded. Previous investigations indicate that under-reporting of food intakes tends to occur during afternoon snacks, dinner, and breakfast (40), suggesting intakes reported at these times of the day require additional attention during a dietary assessment. It has been identified that factors, such as lack of physical exercise and substance use may impact dietary recall (41). For individuals with severe mental health symptoms, food-frequency questionnaires, brief dietary assessment instruments, food image assessments, and wearable cameras may be helpful (41). However, further research is needed to ascertain how accurate these alternatives are in populations with mental health concerns. Ongoing investigations of under-reporting related to mental health status are needed to examine whether the findings observed in this study occur across different subpopulations that include those at different life stages, such as children (42). Furthermore, predictive modeling that can examine a number of factors will better ascertain the relationship between perceived mental health and energy under-reporting. Finally, it is recommended that in large-scale nutrition epidemiology studies, a proportion of the participants experiencing good and poor mental health should be selected and their dietary intake results validated by employing methods such as alternative dietary assessment, examining nutritional status (e.g., anthropometric measures), and measuring nutrition-related biomarkers (43–45).

### Limitations

Although the Goldberg cutoffs are less accurate than objective methods, such as the use of doubly labeled water biomarkers to reference EI, they are considered appropriate for energy under-reporting classification (5). To better identify food energy under-reporters, detailed information on occupation and leisure activity to derive subject-specific physical activity levels to evaluate individual EI should be used. The inflation of the type I error rate from multiple statistical testing may have overestimated the impact that poor mental health has on reporting of energy-adjusted nutrient intakes. Due to limited sample size within groups stratified by age and sex and limitations of variables available in the CCHS dataset, other factors, such as eating behavior (e.g., eating restraint), social desirability, dieting, body image, and race/ethnicity (4, 5, 46), which may mediate or moderate the relationships between mental health state and dietary intakes, could not be assessed. Finally, it has been reported elsewhere that individuals experiencing depression have lower total energy expenditure (47), which raises questions about how food energy under-reporting may be defined in those with poor mental health.

## Conclusions

The report of energy-adjusted nutrient intakes tends to differ among those defined as food energy under-reporters reporting poor and good mental health. This suggests that the mental health state needs to be accounted for when dietary intake assessments are undertaken. This is particularly critical given that diet is becoming increasingly recognized as both a prevention and an intervention target to support mental health (48–50). Future research is needed to discern if deviations in energy-adjusted nutrient intake by mental health state among food energy under-reporters may be attributed to differences in the accurate reports of food intakes or a function of measurement error.

## Data Availability Statement

The data analyzed in this study is subject to the following licenses/restrictions: The data used is from Statistics Canada. Data is secured by Statistics Canada and permission to share data publicly cannot be granted as it might compromise patient confidentiality or participant privacy. The Canadian Community Health Survey data for this analysis was collected by Statistics Canada (third party data). Details about how to access Statistics Canada data are available at: https://www.statcan.gc.ca/eng/rdc/index. Researchers who have been sworn in as ‘deemed employees' of Statistics Canada can access the confidential microdata files for approved projects through Statistics Canada's Research Data Centres (RDCs). The confidential microdata files contain information collected during the survey, derived variables, and the Bootstrap weights used to calculate the exact variance. Requests to access these datasets should be directed to Statistics Canada: https://www.canada.ca/en/health-canada/services/food-nutrition/food-nutrition-surveillance/health-nutrition-surveys/canadian-community-health-survey-cchs.html.

## Ethics Statement

Ethical review and approval was not required for the study on human participants in accordance with the local legislation and institutional requirements. Written informed consent to participate in this study was provided by the participants' legal guardian/next of kin.

## Author Contributions

KD submitted the project proposal that included the research plan to Statistics Canada for approval to access the secure data. KD and LG analyzed the data. KD and VA drafted the manuscript. All authors read and provided edits on manuscript drafts and approved the final manuscript.

## Funding

This project was partially supported by Fulbright Canada funds.

## Conflict of Interest

The authors declare that the research was conducted in the absence of any commercial or financial relationships that could be construed as a potential conflict of interest.

## Publisher's Note

All claims expressed in this article are solely those of the authors and do not necessarily represent those of their affiliated organizations, or those of the publisher, the editors and the reviewers. Any product that may be evaluated in this article, or claim that may be made by its manufacturer, is not guaranteed or endorsed by the publisher.
