# Supplementary material for: Lower Energy-Adjusted Nutrient Intakes Occur Among Food Energy Under-Reporters With Poor Mental Health
Source: Front Nutr. 2022 Aug 8;9:833354. doi: 10.3389/fnut.2022.833354 (PMC9393501; doi:10.3389/fnut.2022.833354)
Supplement: Supplementary file 1 [file Data_Sheet_1.pdf]

Figure S1: Energy adjusted macronutrient intakes that significantly differed by mental health state[a]

Figure 1Sa: Men 31-50 years (median intakes – fibre g/1000 kcal; carbohydrates & protein % of total calories)

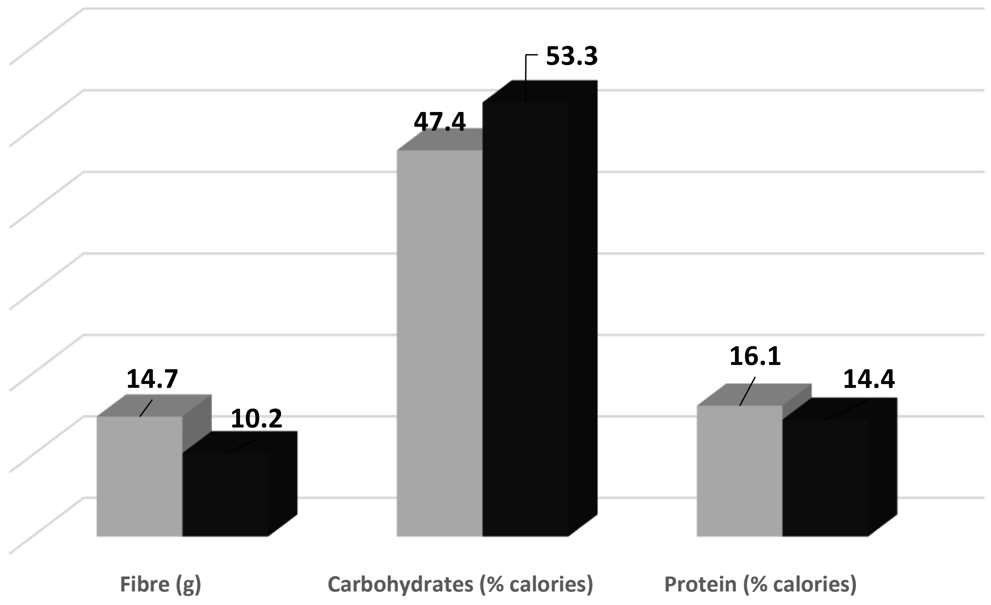

Figure 1Sb: Women 31-50 years (median intakes - fibre g/1000 kcal; protein % of total calories) & 51-70 years (median intakes - fibre g/1000 kcal)

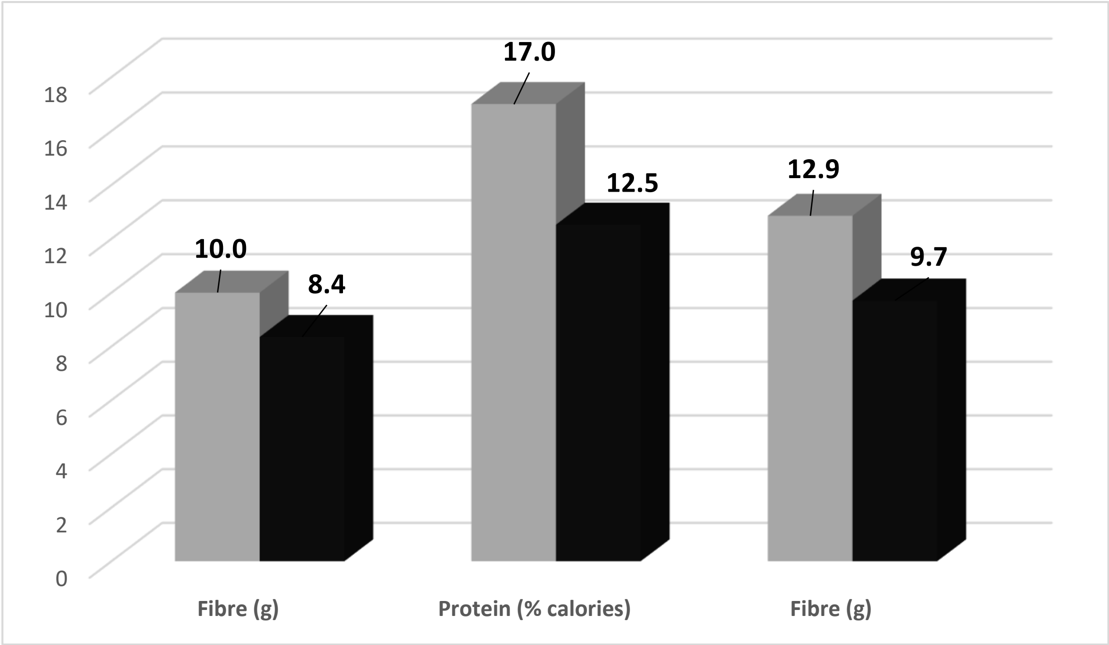

Good Mental Health   Poor Mental Health

g=grams; kcal = kilocalories; [a]All energy adjusted macronutrient intake differences at $p<0.05$

Figure S2: Energy adjusted vitamin intakes that significantly differed by mental health state[a]

Figure 2Sa: Men, 20-30 years (median intakes – vitamins B_2 & C mg/1000 kcal)

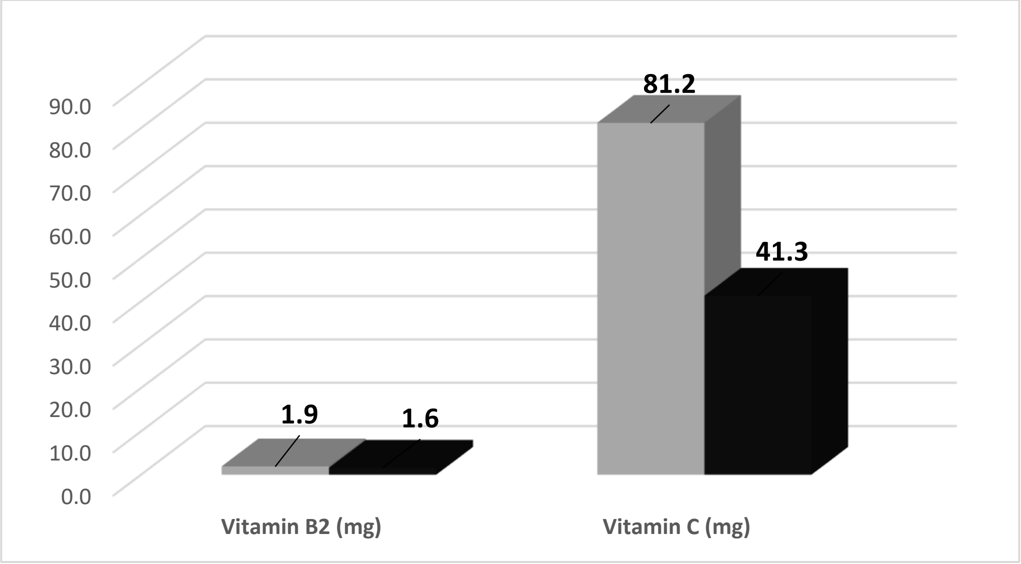

Figure 2Sb: Men, 31-50 years (median intakes - vitamins B_1, B_2, B_3, B_6 mg/1000 kcal; vitamins B_{12} (mcg/1000 kcal) & B_9 (mcg DFE/1000 kcal)

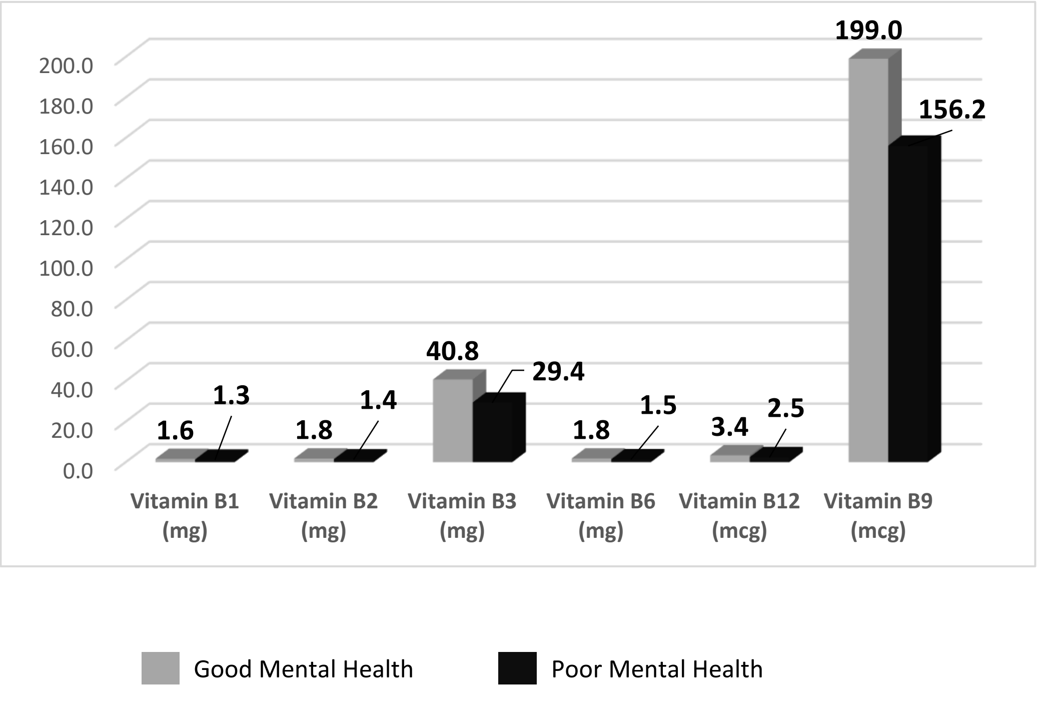

DFE = dietary folate equivalents; kcal = kilocalories; mg = milligrams; mcg = micrograms

Figure S2: Energy adjusted vitamin intakes that significantly differed by mental health state[a] /cont'd

Figure 2Sc: Men, 51-70 years (median intakes – vitamin A RAE/1000 kcal; vitamin D mcg/1000 kcal)

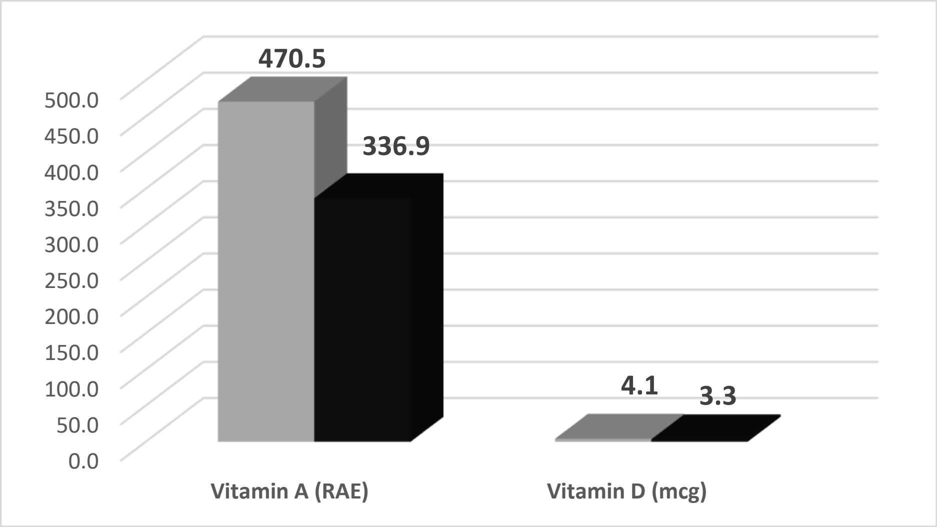

Figure 2Sd: Women, 14-19 (median intakes - vitamin A RAE/1000 kcal) & 20-30 years (median intakes - vitamin B_6 & C mg/1000kcal)

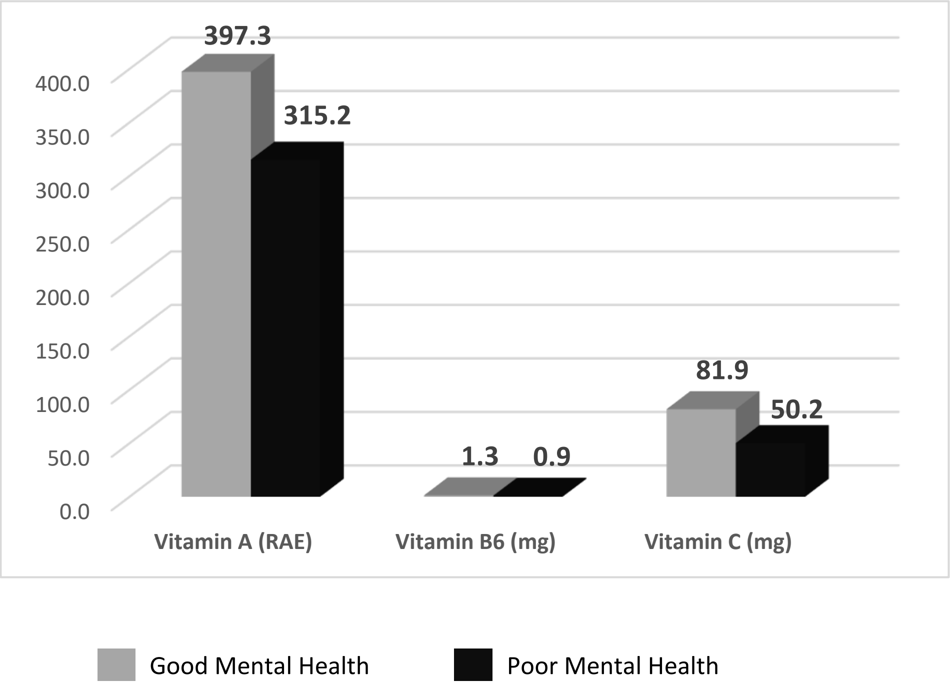

kcal = kilocalories; mg = milligrams; mcg = micrograms; RAE = retinal activity equivalents

Figure S2: Energy adjusted vitamin intakes that significantly differed by mental health state[a] /cont'd

Figure 2Se: Women, 31-50 years (median intakes – vitamins A RAE/1000 kcal, B_3 mg/1000 kcal, & vitamin B_9 mcg DFE/1000 kcal)

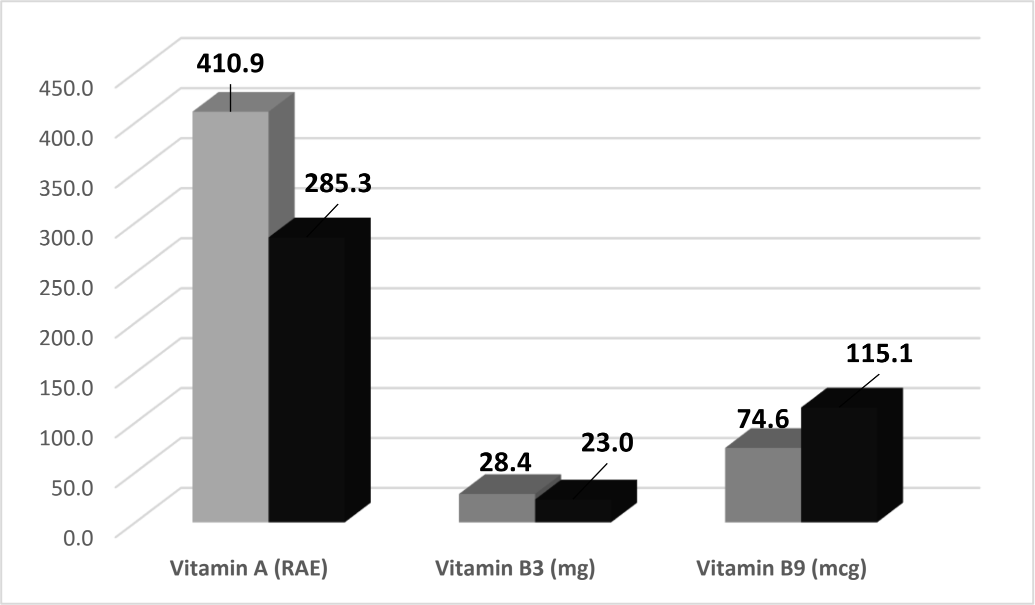

Figure 2Sf: Women, 51-70 years (median intakes – vitamins A RAE/1000 kcal, B_1, B_2, B_3, B_6, C mg/1000 kcal; vitamin B_9 mcg DFE/1000 kcal; vitamins B_{12} & D mcg/1000 kcal)

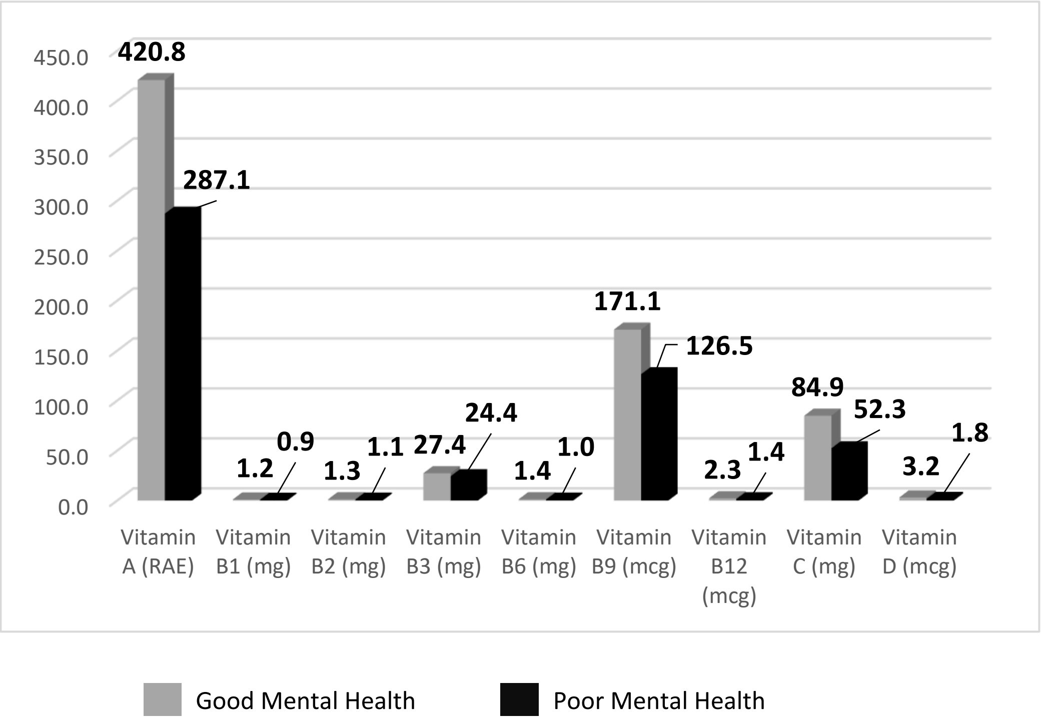

DFE = dietary folate equivalents; kcal = kilocalories; mg = milligrams; mcg = micrograms; RAE = retinal activity equivalents; [a]All energy adjusted vitamin intake differences at $p<0.05$

Figure S3: Energy adjusted mineral intakes that significantly differed by mental health state[a]

Figure 3Sa: Men, 20-30 (median intakes - calcium & zinc mg/1000 kcal) & 51-70 years (median intakes - calcium mg/1000 kcal)

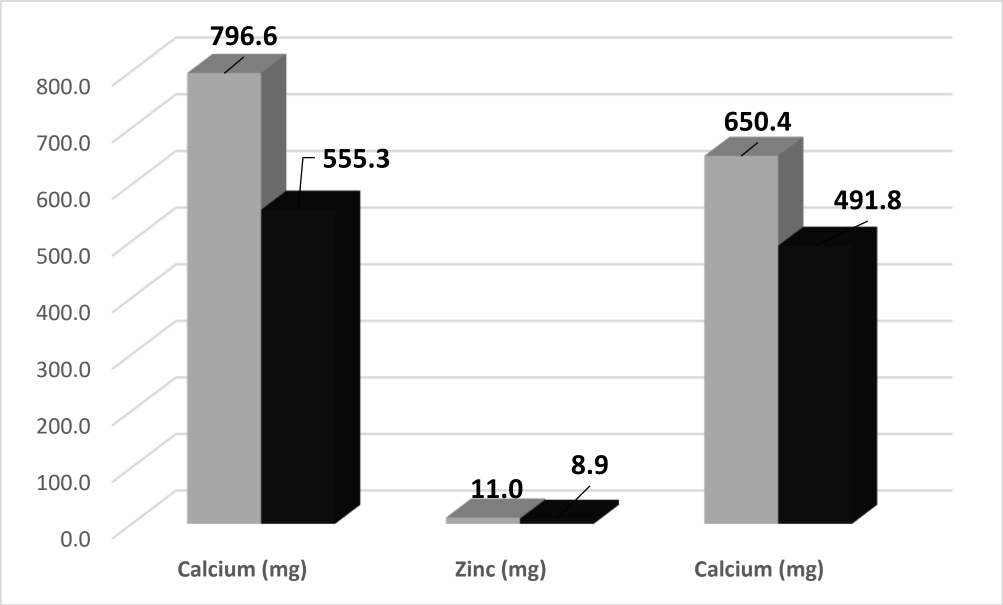

Figure 3Sb: Men, 31-50 years (major minerals: median intakes - calcium & potassium mg/1000 kcal)

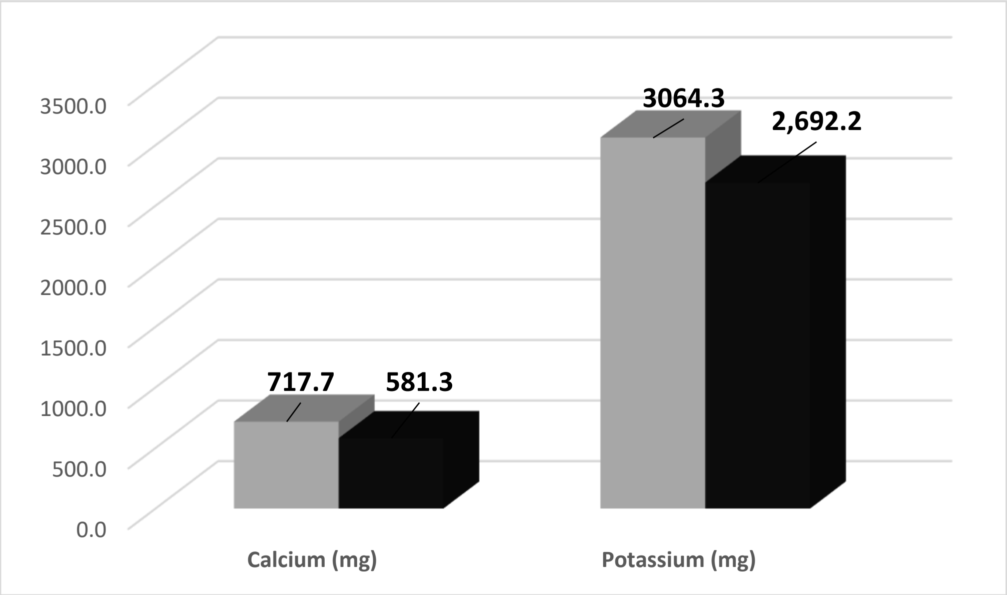

Good Mental Health   Poor Mental Health

kcal = kilocalories; mg = milligrams

Figure S3: Energy adjusted mineral intakes that significantly differed by mental health state[a] /cont'd

Figure 3Sc: Men, 31-50 years (trace minerals: median intakes - iron, magnesium, & zinc mg/1000 kcal)

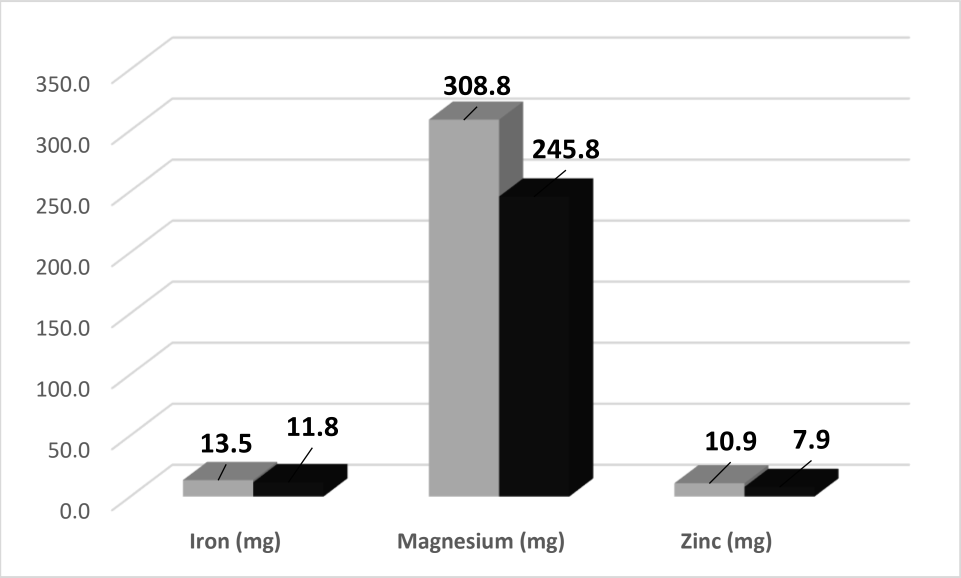

Figure 3Sd: Women, 20-30 (major minerals: median intakes - calcium mg/1000 kcal) & 31-50 years (major minerals: median intakes - phosphorus & potassium mg/1000 kcal)

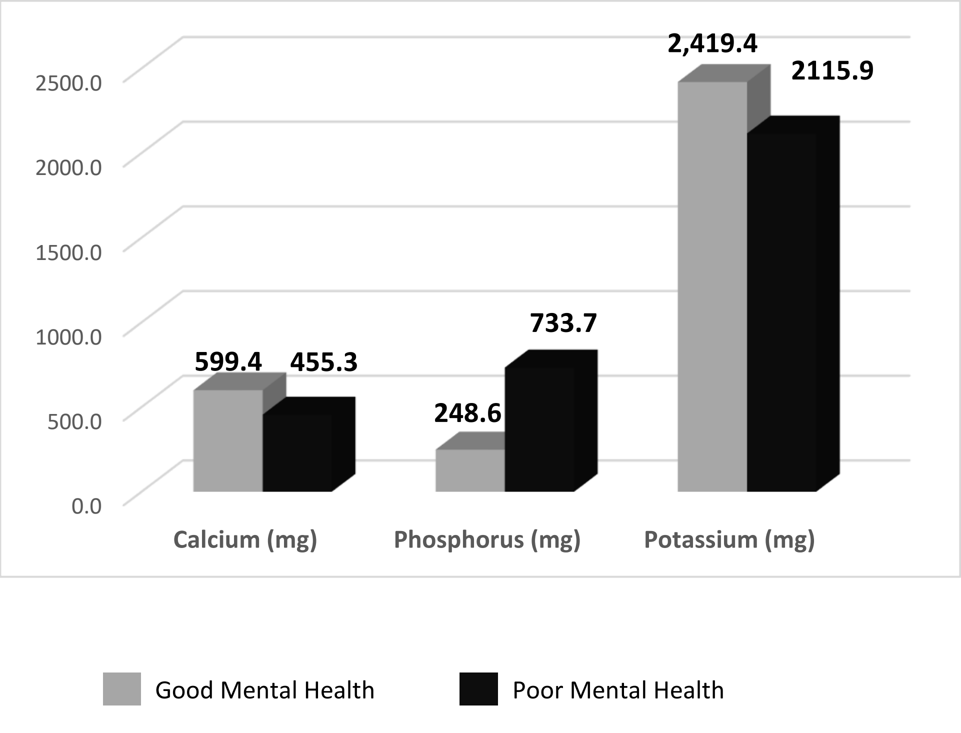

kcal = kilocalories; mg = milligrams

Figure S3: Energy adjusted mineral intakes that significantly differed by mental health state[a] /cont'd

Figure 3Se: Women, 51-70 years (major minerals: median intakes - calcium, phosphorus, potassium, & sodium mg/1000 kcal)

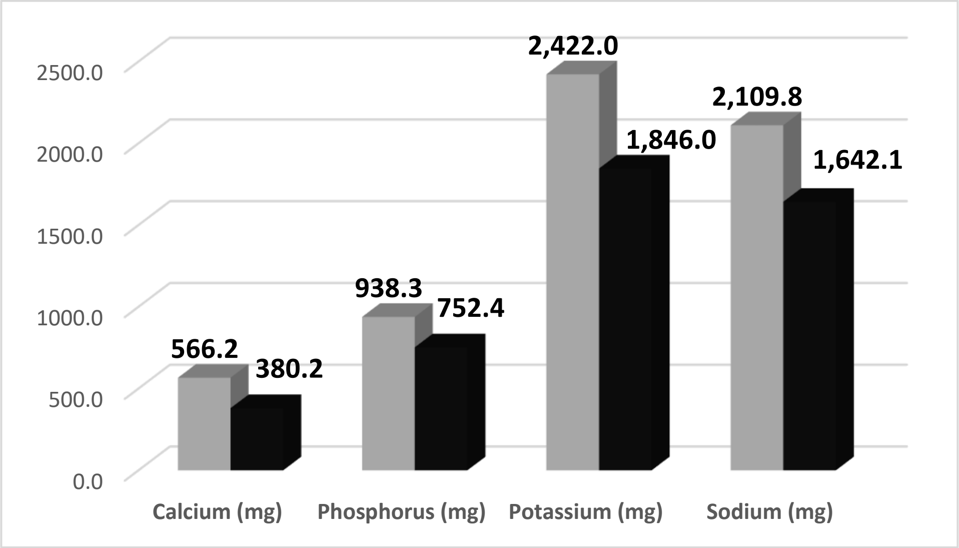

Figure 3Sf: Women, 51-70 years (trace minerals: median intakes - magnesium & zinc mg/1000 kcal)

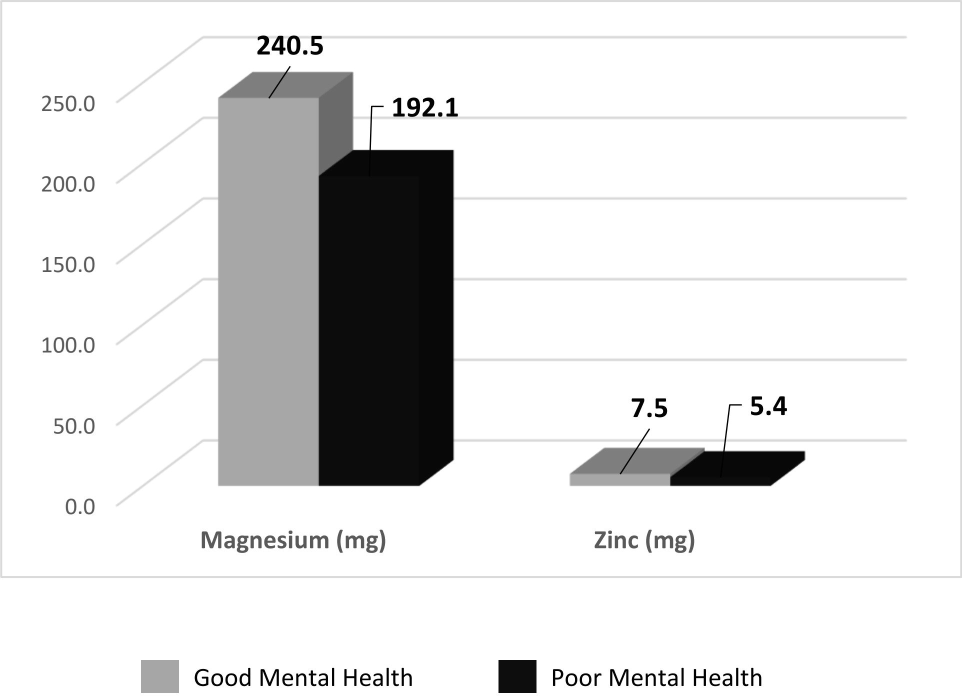

kcal = kilocalories; mg = milligrams; [a]All energy adjusted dietary mineral intake differences at p<0.05
